# VLC Network Design for High Mobility Users in Urban Tunnels

**DOI:** 10.3390/s22010088

**Published:** 2021-12-23

**Authors:** Edmundo Torres-Zapata, Victor Guerra, Jose Rabadan, Martin Luna-Rivera, Rafael Perez-Jimenez

**Affiliations:** 1 Institute for Technological Development and Innovation in Communications(IDeTIC), Universidad de Las Palmas de Gran Canaria, 35001 Las Palmas, Spain; vguerra@idetic.eu (V.G.); jrabadan@idetic.eu (J.R.); rperez@idetic.eu (R.P.-J.); 2 Physics School, Universidad Autonoma de San Luis Potosi (UASLP), San Luis Potosi 78295, Mexico; mlr@fciencias.uaslp.mx

**Keywords:** visible light communications, vehicular communication, handover, MAC/PHY simulation

## Abstract

Current vehicular systems require real-time information to keep drivers safer and more secure on the road. In addition to the radio frequency (RF) based communication technologies, Visible Light Communication (VLC) has emerged as a complementary way to enable wireless access in intelligent transportation systems (ITS) with a simple design and low-cost deployment. However, integrating VLC in vehicular networks poses some fundamental challenges. In particular, the limited coverage range of the VLC access points and the high speed of vehicles create time-limited links that the existing handover procedures of VLC networks can not be accomplished timely. Therefore, this paper addresses the problem of designing a vehicular VLC network that supports high mobility users. We first modify the traditional VLC network topology to increase uplink reliability. Then, a low-latency handover scheme is proposed to enable mobility in a VLC network. Furthermore, we validate the functionality of the proposed VLC network design method by using system-level simulations of a vehicular tunnel scenario. The analysis and the results show that the proposed method provides a steady connection, where the vehicular node is available more than 99% of the time regardless of the number of vehicular nodes on this network. Additionally, the system is able to achieve a Frame-Error-Rate (FER) performance lower than 10^−3^.

## 1. Introduction

In the last decade, the use of the Internet has improved, simplified, and brought new services to many sectors of the economy. In the specific case of the vehicular industry, we expect that Internet-connected vehicles will enable safer driving and enhance passenger experiences. With the connected vehicle market becoming the standard for new cars, they will require the capability to receive real-time information from other vehicles and the roadside infrastructure provided by local authorities. As a consequence of this demand, several communication technologies have been developed. Vehicle Safety Communications (VSC) has defined safety applications to work under the Dedicated Short Range Communication (DSRC) technology [1]. This active vehicle-based safety system has been tested, standardized, and approved by the United States Department of Transportation (USDOT) and some principal car manufacturers such as Toyota, Ford, GM, etc., [2]. Using DSRC, the vehicle reports its status and position continuously at a minimum frequency of 10 Hz. It ensures that the vehicle does not have any issue and that it can be located in case of an emergency. Moreover, it can receive a safety message to prevent collisions or any other possible risk [3].

Other technologies have emerged to support service under adversary conditions for DSRC. Visible Light Communication (VLC) is a potential alternative, which can take advantage of the existing street lighting infrastructure and vehicle lamps to reduce implementation and operation costs [4,5]. VLC enables data transmission in the visible light spectrum (from 380 nm to 780 nm) using solid-state lighting. It modulates optical signals by adjusting the power at high-frequency intervals to be undetected by the human’s eyes. The reception can be possible using photodiode-based receivers or even image sensors. This technology is also recognized as a green technology that does not interfere with radio-frequency-based communications.

The incorporation of VLC in vehicular scenarios has been studied for several years. Vehicular communication systems require wireless data transmission between vehicles (V2V), vehicle-to-infrastructure (V2I/I2V), or both simultaneously. In all these cases, the communication channel is the most critical problem, which has been characterized and modeled to understand its limitations. These channels have been explored following the classical Monte Carlo simulation approach [6,7,8,9], which provides accurate estimations of the channel characteristics such as the channel gain or its bandwidth. Other works study practical measurements to characterize the link and help to get the impact in the VLC communication from different external agents in outdoor environments. Furthermore, the different weather phenomena have been studied before such as snow, light interference, fog, raining, dust storms, among others others have been studied in [10,11,12,13,14]. During the design of the Vehicular VLC network is important to consider that the uplink and downlink will be asymmetrical in most cases. Further to this, the signal propagation depends on the radiation pattern of the lamp, which is different even in the V2V case where the vehicle from behind uses a headlamp while the car in front of it transmits with a taillamp. These factors create an unequal link range between the nodes, which leads to some open issues [15]. On the other hand, the VLC channel is more robust to variations than the DSRC channel. It has a longer coherence time and it is not highly affected by Doppler effects [16]. Moreover, the average link duration depends on the type of street, highway, urban street, or rural road. In urban areas, the V2V link lifetime is around 6 s [17]. Although VLC does not substitute DSRC, it will operate when traditional RF technologies can not provide this service. For example, the vehicular tunnel is a scenario where RF technologies degrade their performance due to multipath propagation and the continuous variations of the channel [18,19].

Previous research works on VLC for vehicular communications have mainly focused on the physical layer aspects of point-to-point communication. Few studies have been done to explore the higher layers issues when deploying a vehicular VLC network. For instance, in V2I cases, the network restructures rapidly, preventing users from staying connected to an AP for more than a few seconds. As consequence, the vehicle reduces its available connection time. This barrier has produced a growing interest in studying handover mechanisms for continuous communication in the vehicular network. The user could tackle this problem by holding the session while moving through the different Road-Side Units (RSUs). The handover process requires the coordination of the APs in the area with upper-layer entities. The handover is a set of steps to detect in advance when the user’s node is on the coverage area bounds. Then, it informs the next AP to prepare resources before the communication link breaks. Depending on the movement of the vehicular node, it can move to a neighbor VLC AP (horizontal scheme) or migrate to another communication technology (vertical handover). The implementation of handover schemes for VLC is still at a very early stage, with only a few works within this open area of research [20,21]. Section 2 provides a more detailed analysis of this problem.

In this paper, we propose a VLC design solution that provides reliable connections for high mobility environments. The main contributions are twofold: firstly, we develop a promising VLC network topology that restructures the uplink connection process to ensure the reliability of the handover decision. These modifications allow increasing the redundancy on the uplink to avoid abrupt interruptions. Then, we introduce a novel handover scheme with a fast resolution protocol that copes with high mobility in VLC based networks. The scheme takes advantage of the predictable channel response variation to trigger the handover process opportunely. Moreover, we create a system-level simulator to validate the proposed solution in a simplified but still accurate representation of a vehicular VLC network. As an example, we evaluate the operation of the VLC network over a realistic vehicular tunnel scenario. Simulation results show that the proposed method allows a vehicle to connect longer times in high mobility scenarios, as much as 99%, regardless of the number of vehicular nodes on the network. Moreover, the FER is used to measure the system reliability after the re-transmission and detection mechanism of the MAC layer. In general, the system can attain FER lower than 10^−3^.

The remainder of this paper is structured as follows. Section 2 presents the comprehensive details of the handover process and an overview of the related works applied to V2I links. A summary of the IEEE 802.15.7 MAC and PHY layers is discussed in Section 3, which provides a framework for our work. Section 4 introduces the proposed system design, it first describes the network topology changes to fasten the handover resolution, and then it explains the proposed handover strategy for this problem. Section 5 describes the evaluation methodology, and Section 6 discusses the obtained results. Finally, we draw the conclusions and future work in Section 7.

## 2. Related Works

VLC technology is still in the early stages of development, in consequence, there are some problems in the superior layers that have not been deeply studied yet. One of the less-studied topics is the mobility process when a user changes from one AP to another. The main drawback of this procedure is the time spent during the association process to initialize a new session. If the vehicular node requires to move across many cells, the continuous cell exchange will limit the communication. Without a mobility scheme, a user’s node has to accomplish some processes to recover service each time it enters a new network. First, it needs to detect the link failure which presents a compromise between sensibility and reliability. If the criteria are too sensitive, the user can disconnect needlessly with a ping-pong effect. On the other hand, resistant detection can hold the node without service for a long time. The second step is scanning the channel looking for a new AP with appropriate connection conditions. When the user’s node has decided on the target AP, it executes an entering protocol. Unfortunately, these actions take a long time and disable the link communication for some seconds.

Although there are several proposed mechanisms in the current IEEE 802.15.7 VLC standard [22] to help with mobility, they are considered basics. These mechanisms include cell arrangements to get an accurate cell location and time distribution to avoid conflicts for users leaving the coverage area. For the localization process, a group of *n* cells emits sequentially a frame to estimate a position, taking a long time. But, this is an unpractical solution for a user with high mobility speed. Moreover, such a standard does not provide a resolution protocol to make the handshake. It also does not give a procedure for estimating when a user moves away from the AP’s coverage area. Thus, the lack of previous studies on this topic represents a potential research gap that needs to be fully explored.

### 2.1. Handover Process

The handover process in the literature is commonly divided into three phases regardless of the communication technology and the handover strategy employed. These phases are information gathering, handover decision, and handover execution. In general, the research works in this area cover only one of these sub-processes.

Information gathering is the sub-process of collecting the input signal to determine when the handover starts. This signal definition depends on the problem to study. For the vertical handover, it requires something that can be measured no matter the propagation medium. So, it uses upper-layer information that reflects the Quality-of-Service (QoS). This can be determined by many aspects such as the total latency, the bandwidth provided, or how reliable is the communication in each path. For example, protocols like Multipath MTCP senses each link’s round-trip latency to determine the best option [23,24]. Additionally, multiple inputs can be computed to get more accurate results analyzing them through a cost function [25,26,27]. It is modeled to see the spent resources considering the number of signaling messages necessary to complete a handover. When the network increases in size, it affects its performance and cost. On the other hand, horizontal handover requires collecting information that reflects the spatial position of the user. It compares the information of multiple APs to determine the closest. One common Physical indicator is the Received Signal Strength (RSS), which considers the proximity to an AP [28,29]. In a complementary way, the Signal-to-Noise Ratio (SNR) or Signal-to-Interference ratio (SIR) can execute the same function. Furthermore, there are some link-layer indicators, such as Bit-Error-Rate and Frame-error-Rate, which can be used to estimate the time to proceed with a handshake process [30,31,32]. This collected information is treated in further stages of the handover process.

The second phase is the application of the signal detection strategy to trigger the handover execution. This phase can use algorithms with fixed or dynamic thresholds, depending on the system’s nature. Additionally, the handover criteria use multiple input and memory statements to improve efficiency. One of the main issues to deal with is the temporal anomalies of the tested signal. For example, optical systems suffer from shadowing effects. These phenomena can incorrectly trigger the handover process. For this reason, the handover-based RSS resolves after a second dwell measure corroborates the failure [33]. In some scenarios, a fixed delay presents a poor performance in the handshake estimation. For example, if a user often moves, the waiting time needs to be shorter. A dynamically dwell can deal better with an outage in these cases. It can consider the frequency that the user requires to do a handover during the last days or how much the received signal changes to adjust the tolerance time [34]. These algorithms require considering the communication signal from neighbors to decide to initialize a horizontal handover. When the signal difference between the APs is relatively small, the system may perform an incorrect estimation. To avoid these fluctuations in the handover decision, the target AP’s signal value needs to surpass the current value with an additional tolerance, which is known as Handover Margin (HOM). When the user has changed the service provider, it cannot return to the previous AP until more robust return conditions have been accomplished. It shapes a hysteresis loop, which prevents the user hops from AP to AP when the link fluctuates [30,35]. There is also a time restriction to avoid multiple handovers in a short period, called Time-to-Trigger (TTT).

The last phase is the handover execution that performs the steps to resolve the mobility. These steps include a set of messages to request system resources and inform all the network elements about the new user localization. It is also necessary to establish the network topology with upper layer modules. Sometimes, the modules require adding links to exchange messages such as X2 in LTE technology. The mobility mechanism can have a hard handover event when the transmission is cut off and immediately a new link is established. It requires the least processing by the network to be imperceptible to the users’ applications. On the other hand, a soft handover event happens when the current and target APs provide service coordinately during the transition phase until the handover process has been resolved. An example of these schemes is Coordinated MultiPoint transmission (CoMP) which has been studied for VLC applications [28]. In this scheme, the current and target APs share a time window to transmit coordinately the exactly optical signal simultaneously. It helps to avoid a communication interruption when the user commute from one AP to the other. This technique requires precise coordination to prevent symbol interference. Other soft handover schemes use unsynchronized transmissions, the packets are sent by both AP, but not at the same time/medium [36].

A significant part of the time spent during the handover resolution is applied in updating external agents about the user location. The network needs to give a new address to the user, so it is necessary to check Duplicate-Address-Detection (DAD) and later upload at Home Address entity the Care-of-Adress (CoA); both actions take significant time. Indoor protocols take a long time, even the fastest version like Fast Handover Mobile IPv6 (FMIPv6) takes over 30 ms [37]. However, in high mobility networks such as vehicular networks, they can not tolerate a long delay in the resolution. Thus, they need to opt for the fastest protocol possible. An approach for this is to redirect the traffic to the new target AP during the handover execution. The user is made aware of the current AP about mobility. Then, the current AP exchanges parameters with the target AP and the mobile user to the start session between them. When the packets are delivered to the previous AP, it buffers them using a tunneling session [38,39,40,41,42]. One of the most common tunneling protocols is MultiProtocol Label Switching (MPLS). The process continues until the handover has been resolved. Proxy Mobile IPv6(PMIPv6) is a protocol based on this technique [43].

Another existing approach is to avoid updating the network configuration to external agents. In these cases, the user identifies its link with an internal address. When the node needs to exchange AP, the resolution is locally known as handover Layer 2 (L2). L2 reduces many processes regarding a typical handover (L3). It only requires the scanning process to determine when it is necessary to move the node, an authentication that involves a local network coordinator, and the re-association signaling message. This scheme avoids updating the node localization to other networks. One protocol that can provide tools to do this action is Hierarchical Mobile IPv6 (HMIPv6) [44]. This model will be detailed further in Section 4.1. The protocols are not mutually exclusive and they can be combined [45].

### 2.2. Handover for Vehicular VLC

Although VLC is emerging as a viable option for vehicular networks, the number of research groups dealing with handover in VLC vehicular scenarios is very limited. In the last years, few works have been published in this area. Murat et al. have studied the channel for indoor scenarios using Jointly Transmission COMP (JT-CoMP) in the two APs [28]. The results show an improvement in the link data rate. They have also evaluated this scheme in vehicular scenarios using the same methodology. As a result, they noticed some problems with its execution. So, they adapted the scheme to decrease the impact of the vehicle speed variations. Their algorithm incorporates dynamic steps on the HOM and TTT [21].

An alternative solution is presented by Jarchlo et al. [46,47]. They create a handover scheme named "Flight" based on link aggregation using Multipath TCP protocol (MPTCP). In this scheme, the vehicle can support two VLC links working at the same time, each one with its own address. One provides communication while the other serves as a backup. The protocol patrols the link status using the ARP message. When the system cannot provide a round trip communication, it faces a link failure, and then it uses the auxiliary link. The route modifications are performed in the Transport layer, where the packet’s destination address is changed. This minimizes the disconnection time. During this period, the vehicle nodes can detect a new AP to establish another backup link [46,47]. The outage time depends on the ARP frequency. For this scheme, the user requires two receivers and two transmitters to hold simultaneously the communication in both links.

A system-level simulation tool provides a common way to validate new handover schemes. Due to the relative novelty of VLC, there are not many software libraries of this technology for this kind of simulator. While some research groups have created some ns3-based modules to evaluate VLC links [48,49], none of them provide official support. However, these modules mostly consider only the link physical layer properties ignoring protocols from the link and upper layers. Some additional research works include evaluations of the multiple access scheme on the standard IEEE 802.15.7 under diverse conditions without evaluating the VLC link [50,51,52].

## 3. Overview of IEEE 802.15.7

To evaluate the proposed handover strategy, we need to consider the impact of its properties in the VLC communication. For instance, the channel access and other mechanisms on the Medium Access Control layer (MAC) can cause latency on the protocol resolution, as a consequence, sometimes the user may not be able to complete the handover. On the other hand, the transmission properties described on the Physical layer (PHY) such as modulation scheme, codification, among other things dictate the link capability and range. For these reasons, this work includes the system’s assessment of several processes and specs from the IEEE 802.15.7 MAC and PHY layers [22]. In what follows, we will describe the most relevant protocols in the MAC layer and the main aspects to consider about the PHY layer.

### 3.1. IEEE 802.15.7 MAC Layer

The MAC layer takes care of the logical decisions from different nodes. Some of the protocols that cover the IEEE 802.15.7 MAC layer are an association of a new user, AP incorporation, transmission schemes, synchronization, and some mobility mechanisms, among other procedures.

**Figure 1:**
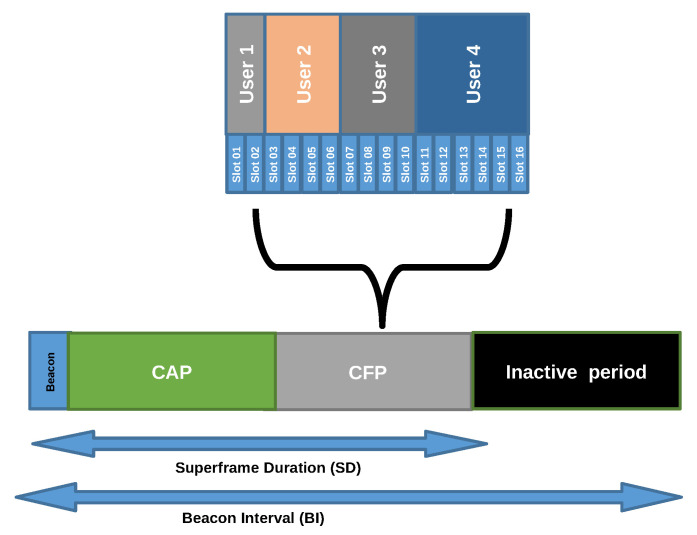
Superframe structure in the IEEE 802.15.7 standard.

In a star topology, the users are ruled by a centralized AP node, called Coordinator. The synchronization of the network nodes is possible by a superframe (SF) structure allowing communication with the network elements. The IEEE 802.15.7 superframe structure, which is shown in Figure 1, is divided into active and inactive periods. The nodes communicate during the active period and remain in sleep mode during the inactive period. The active interval is further divided into two parts, Contention Access Period (CAP) and Contention Free Period (CFP). The superframe begins when the coordinator transmits a beacon frame to synchronize the users. If a user loses this beacon frame, he can not perform any action until a new beacon is received. Furthermore, the node with a long unsynchronized time needs to join to new APs. The duration of the superframe is known as Beacon Interval (BI) which depends on the optical clock (OC) frequency, the number of slots per frame, the Beacon Order (BO), and the number of the optical clock in the aBaseSlotDuration usually equal to 60 OC [22]. Equation (1) defines the time duration for BI

(1) $$BI=aBSFD\times 2^{BO}$$

with

(2) aBSFD=aNSFS×aBSD

where

- aBSFD=aBaseSuperFrameDuration
- aNSFS=aNumSuperFrameSlot
- aBSD=aBaseSlotDuration

**Figure 2:**
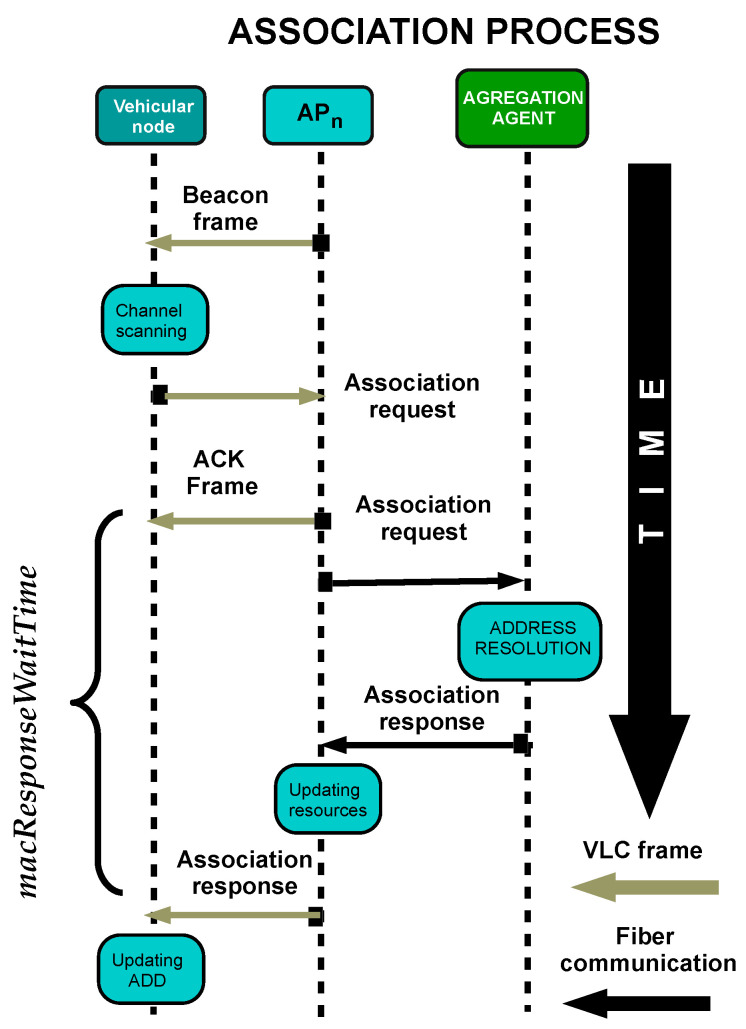
Diagram of the association process in the standard 802.15.7.

One vital aspect to consider is the time to recover communication service when a handover fails determined by the following association protocol. The association protocol can be seen in Figure 2 First, the user scans the optical channel searching for the best AP available, sensing any beacon frame. When the user detects the first beacon, it starts a waiting period of $2^{n}+1$ BI. Then, the user decides the best option by choosing the strongest Received Signal Strength (RSS). It transmits the Association Request frame using the standardized format. Next, the coordinator needs to send an ACK frame to inform the user that the petition is in progress. So, it does not try to ingress again for a macResponseWaitTime waiting period until its petition has been resolved. Later, the coordinator starts the registration of the user with multiple entities of the Core Network. When the validation process has been completed, the coordinator informs the user with an Association Response.

**Figure 3:**
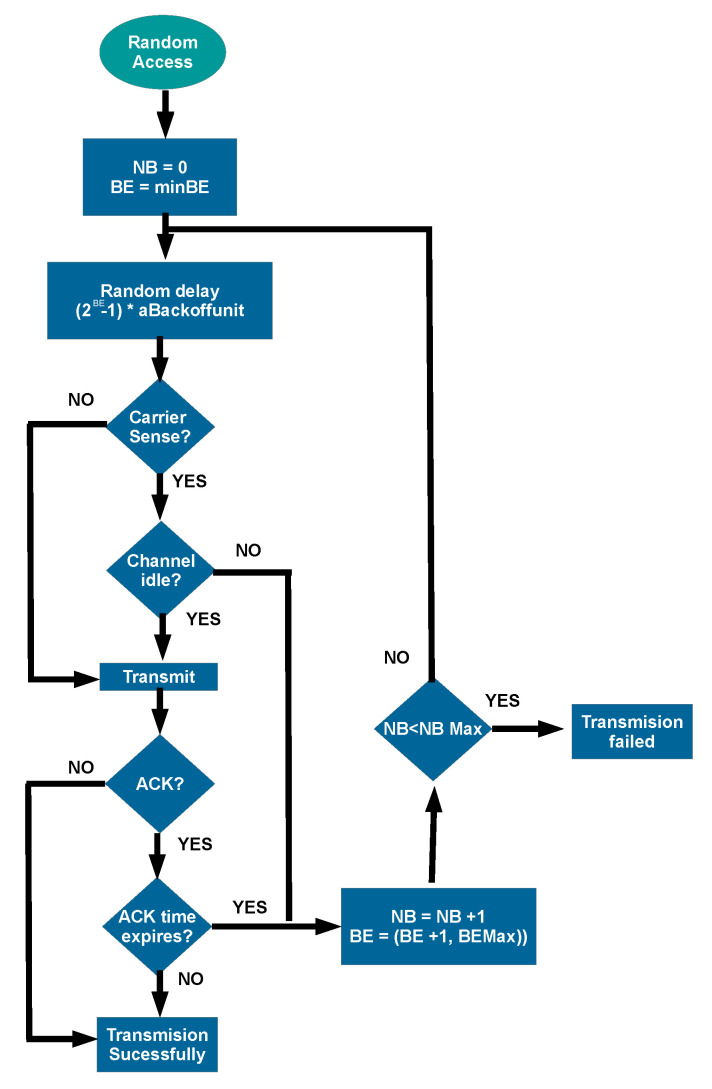
The transmission algorithm during CAP in standard IEEE 802.15.7.

The handover procedure execution time can be delayed by the delivery of the request and other signaling messages. It depends on the channel access controls used. Moreover, the VLC transmission performs Carrier Sense Multiple Access with Collision Avoidance (CSMA/CA). In this scheme, transmissions require a waiting period, known as Contention Window (CW), to allow multiple users to share the medium. The duration of CW is a random variable with a uniform distribution that depends on BackoffExponent (BE) and aUnitBackOffPeriod, as it can be seen in Equation (3). The parameter BE is related to the number of retransmissions, while aUnitBackOffPeriod is a base number of optical clocks. Before transmitting, the node senses whether the channel is idle or busy. When the channel is busy, the transmission is reprogrammed for a longer time. If the channel is free, the node transmits the frame and waits for an ACK to confirm the correct delivery. When it has failed, BE, and the number of backoffs (NB) increase. After several failed transmissions, the frame is discarded when NB is higher than a limit, and all the counters are reset. For a better understanding, Figure 3 shows a flowchart of this transmission scheme.

(3) $$CW=(2^{BE}-1)\cdot aUnitBackoffPeriod$$

### 3.2. IEEE 802.15.7 Physical Layer

A VLC system consists of a transmitter based on lighting LED sources, a propagation channel ranging from 380 to 780 nm in the electromagnetic spectrum, and light-receiving elements such as photodiode detectors or image sensors. Next, we summarize the relevant aspects of the IEEE 802.15.7 PHY layer for the optical wireless communication standard.

First, a LED-based VLC system cannot encode information in the phase or amplitude of the light signal. Instead, it uses Intensity Modulation/Direct Detection (IM/DD) schemes to transmit data. But the presence of other light sources in the same medium plays a major role due to the carrier limitation. Despite this, some recent efforts on multiple access-based modulation schemes show the potential to alleviate this restriction. An example of such schemes includes Color-Shift Keying (CSK) modulation, which allows multiple access through the variation of the color emitted by the red, green, and blue light-emitting diodes (RGB LEDs). These schemes are, however, limited to only three different bands (one per color). Other popular modulations schemes such as QAM, OFDM, or other variants as DCO-OFDM, have also been adapted to deal with these types of restrictions [53].

The first release of the IEEE 802.15.7 standard defined three PHY layer types (I, II, and III) which were grouped by data rate. More recently, amendments to this standard added three more PHY modes (IV, V, and VI) for low-rate photodiode communication and optical camera communications (OCC). While PHY I is intended for outdoor applications with data rates from 11.67 kbps to 266.6 kbps, PHY II is designed for indoor usage with moderate data rate applications from 1.25 Mbps to 96 Mbps, and PHY III for applications using multiple optical sources with data rates from 12 Mbps to 96 Mbps. The modulation schemes used in PHY I and II modes are on-off keying (OOK) and variable pulse position modulation (VPPM), and for PHY III is color-shift keying (CSK) that allows multiple light sources and detectors. On the other hand, PHY types IV, V, and VI incorporate multiple modulation schemes that target a broad range of OCC applications. A detailed description of the OWC PHY specification modes can be found within the IEEE 802.15.7 m standard [22]. Additionally, the standard also includes two codifications stages. The first is Run-Length Limited(RLL) which uniforms the illumination level. The second is Reed-Salomon codification which decreases the errors on the transmission.

Another relevant point is the channel effects in the communication. On the one hand, the received signal power decreases quickly as the distance grows. At the same time, the scenario reflections create multi-path propagation effects that can limit the maximum data rate. In general, VLC channel models are composed of line-of-sight (LOS) and non-line-of-sight (NLOS) contributions. While LOS can be calculated with a closed formula, NLOS depends on the scenario layout. In this way, the Modified Monte-Carlo simulations (MMC) method with ray-tracing [54] is an alternative tool to obtain the impulse response of the VLC channel. MMC considers the light radiation patterns to generate random rays and emulates the light propagation in the scenario. Each rebound has a deterministic LoS contribution to reducing the number of rays necessary to get a good approximation of the CIR. The channel information provides the power and bandwidth restrictions at determined moments and positions. At the same time, the signal propagating in the channel affects the Signal-to-Noise Ratio (SNR) and increases the bit error rate (BER). Particularly, the power SNR at the output of a PIN photodiode can be formulated as

(4) $$SNR=\frac{{\left(P_{tx}H\left(0\right)R_{\lambda }\right)}^{2}}{\sigma_{th}^{2}+2q\left(P_{tx}H\left(0\right)R_{\lambda }+i_{d}+i_{b}\right)B}$$

The three main noise sources are included in this equation. The first source is Johnson noise $\sigma_{th}^{2}$ generated by the components of Trans-impedance amplifier electrical noise. It is also known as thermal noise. Then, there is the shot noise induced by the signal and ambient light. It needs to include the dark current component $i_{d}$ of the photodetector. In Equation (4), $R_{\lambda }$ is the responsivity of the receiver, $\sigma_{th}^{2}$ is Johnson noise, *q* is the electron charge, $i_{d}$ is the receiver’s dark current, $i_{b}$ is the background-induced current, and *B* is the noise bandwidth of the receiver. In this noise model, the transmitted signal decreases its initial power, $P_{tx}$, where the decrement ratio can be calculated by the DC channel gain H(0), that is the sum of all LOS and NLOS contributions of the transmitted signal in the scenario. Each one of the typical values can be seen in Table 1. However, it is important to clarify that Johnson noise value depends on Equation (5). Where η is the Fixed Capacitance, *G* is the open-loop voltage gain, Γ is the FET transistor noise factor, $T_{k}$ is the environment temperature, *k* Boltzmann’s Constant and $I_{2}$ and $I_{3}$ are Noise Bandwidth Factor. The parameters and noise model were based from [49] that they validated experimentally.

(5) $$\sigma_{th}^{2}=\frac{8\pi kT_{k}}{G}\eta AI_{2}B^{2}+\frac{16\pi^{2}kT_{K}\Gamma }{g_{m}}\eta^{2}A^{2}I_{3}B^{3}$$

## 4. Proposed VLC Network for High Mobility

In this section, we introduce a comprehensive solution to ensure a seamless connection to vehicular VLC users. The system aims to operate as support or backup for DSRC based technologies. This solution modifies the typical VLC network star topology to increase uplink reliability for a vehicular tunnel scenario. Further to this, it includes a novel handover strategy that covers the signal detection and execution phases. The subsections below describe the proposed VLC network design for high mobility.

### 4.1. VLC Network Topology

**Figure 4:**
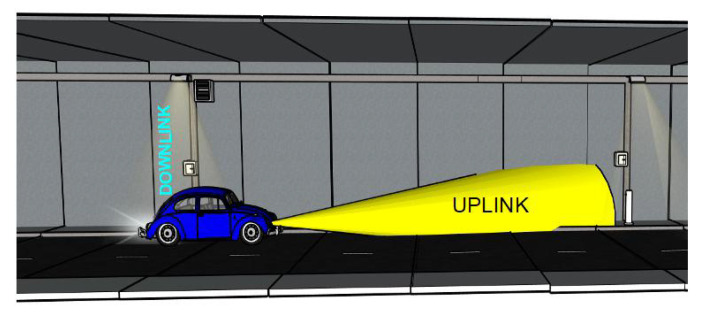
Graphical representation of the vehicular VLC communication system in V2I mode.

The network communication layout comprises a group of APs built-in the illumination system along the entire tunnel, which acts as RSUs. Each vehicular node has a VLC module that uses the headlamps as a transmitter and a photodiode-based receiver on the vehicle’s hood. A full-duplex VLC system is assumed between a vehicular node and the APs. Figure 4 depicts the deployed vehicular VLC network scenario in which the tunnel roadside infrastructure provides coverage for the vehicular nodes. The AP’s receiver is at 13 meters (m) forward its transmitter and a half meter from the ground level. This receiver’s position is determined based on our previous analysis presented in [55,56], where the channel propagation is studied for both uplink and downlink. The results show that the AP benefits to separate RX from the rest of its hardware to get a LOS contribution from the vehicular node.

**Figure 5:**
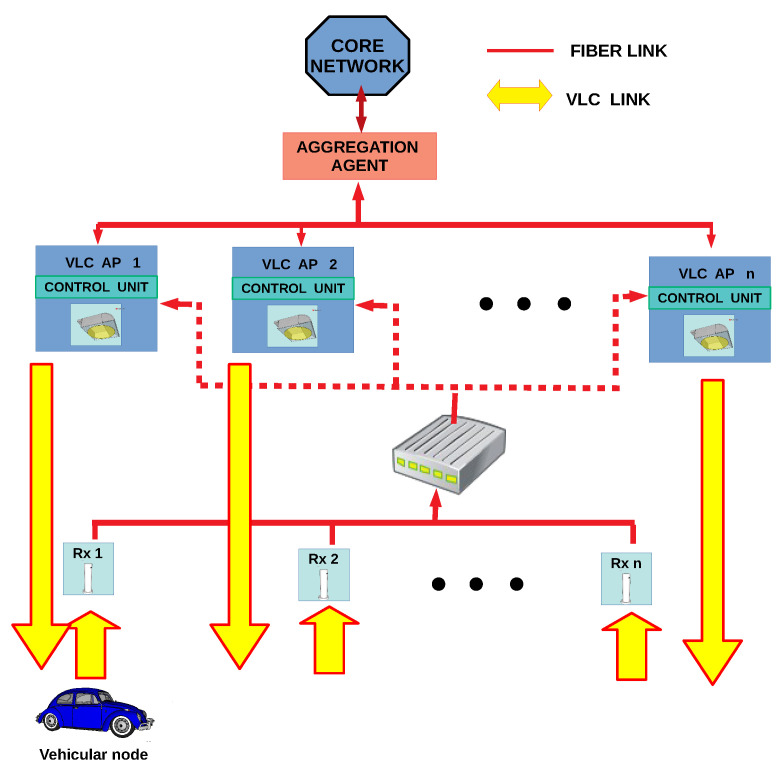
2.5 Layer topology diagram.

Due to the AP’s receiver location, the uplink’s power signal increases gradually. But suddenly, it suffers a drastic power reduction. Under this situation, an untimely handover initialization can break the uplink easily. So, it is necessary to increase the redundancy in this link to avoid abrupt interruption in the communication. As a partial solution, a new topology was presented in [57]. The topology extends the uplink range by integrating all the infrastructure’s receivers in a new sublayer named “2.5”. For this case, the receivers work as standalone devices connected by fiber to a common switch, which acts as an intermediary between receivers and the APs. Each receiver performs the optical signal demodulation and decodification to recover the binary information. Then, they send the information to the switch which checks the OWPAN-ID address to know the destination and redirect it. Finally, the AP receives the frame information and manages the channel access and other processes in the communication. This topology aims to extend the uplink range by increasing the time to resolve a handover. It allows the AP to receive a VLC frame when the vehicular node has passed its nearest receiver. It is because the switch redirects the frame using its header information allowing the collection of frames from any receiver. There are some cases when two or more receivers can receive successfully the same frame. If this situation happens, the switch will eliminate the redundant frames of the network. It allows the system to operate when a handover has not been initialized opportunely without disrupting the service. Figure 5 displays the diagram of this 2.5 layer topology.

Furthermore, the APs are managed and coordinated by using an Aggregation Agent (AA). This topology follows the structure of IoT Device Management protocol (IDMP) which includes self-configurable devices [58]. Also, AA has to deal with the main actions during the mobility process. Internally, it has two virtual modules: Mobility Anchor Point (MAP) and Handover Manage Entity (HME). MAP keeps a database of the current user location in the network. So, the users are tracked using a local ID address following the HMIPv6 scheme. It also helps the user to retain its IP Address so that the mobility process will take less time. On the other hand, HME collects the information from other network entities to decide when starting a handover. The decision algorithm and the gathered information process are described in Section 4.2.

**Figure 6:**
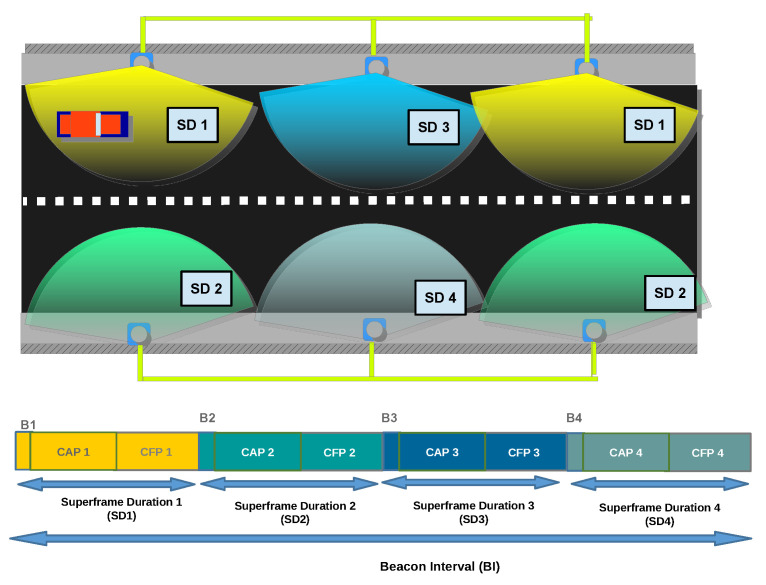
Cells distribution over the evaluation scenario (**top**), and cells distribution on the superframe structure (**bottom**).

Another system design consideration is the distribution of the cells. In the IEEE 802.15.7 standard, there is a superframe configuration to support the user’s mobility. In this configuration, multiple cells work coordinately transmitting the beacon at the same time and using a common CAP. The CFP is assigned per cell sequentially to avoid interruptions. In contrast, in our proposed system design, each site uses the superframe configuration for a single site. BI is 2 ms long and four times SD with a Beacon Order of 7 and SuperOrder of 5. Thus, each AP is active just a quarter of the time. The rest of the time is used in turns by neighbors APs. So, the SD shifts on the time to work as Time Division Multiple Access (TDMA), see Figure 6. These actions allow coexistence between contiguous APs and the user, reducing the system throughput as commented before.

### 4.2. Handover Strategy

The new handover approach is based on SNR information from downlink and uplink channels. The downlink SNR is measured when the AP transmits a beacon frame, and the uplink SNR by sending pilot frames periodically every 5 ms. In our previous work [57], the system design (topology configuration) was evaluated by triggering the handover process using only uplink information. However, this information is highly affected by the lateral displacements of the vehicle that contaminates the estimation. As a consequence, the system does not perform the handover on time, and the vehicular node loses the link. Therefore, in this paper, we formulate and solve a new handover decision scheme based on the SNR variations in both uplink and downlink channels. To better cope with the handover sensitivity, we use two different criteria to trigger the handover, one over the downlink channel and another for the uplink channel. The handover will initiate if at least one has fulfilled its conditions.

**Figure 7:**
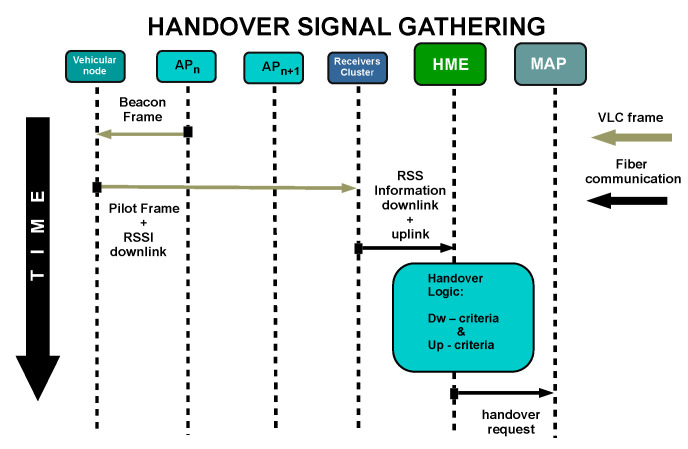
Signal gathering stage.

The handover scheme is a three-stage process. The first stage is the signal gathering process which is depicted in Figure 7. During this stage, the current site $AP_{n}$ transmits a beacon frame to synchronize the users. When the vehicular node receives this signal, it estimates the instantaneous SNR of the downlink defined by $P_{DL}\left(t\right)$. Then, this node broadcasts a periodic pilot frame that includes the measured $P_{DL}\left(t\right)$. This pilot frame arrives at multiple AP receivers, which individually estimate the instantaneous SNR of the uplink channel, i.e., $P_{UL}^{n}\left(t\right)$ for $AP_{n}$, $P_{UL}^{n+1}\left(t\right)$ for $AP_{n+1}$, etc. The links information is then shared with the network switch in a single frame. After that, the information is sent to the HME to analyze if it is necessary to initiate a handover process. In our scheme, this entity only considers the instantaneous SNR values from the current site, $P_{DL}\left(t\right)$, and next site, $P_{UL}^{n+1}\left(t\right)$, for the decision stage. Additional benefits could be gained by incorporating the extra SNR inputs from other receivers, however, it needs a further study that is out of the scope of this paper.

**Figure 8:**
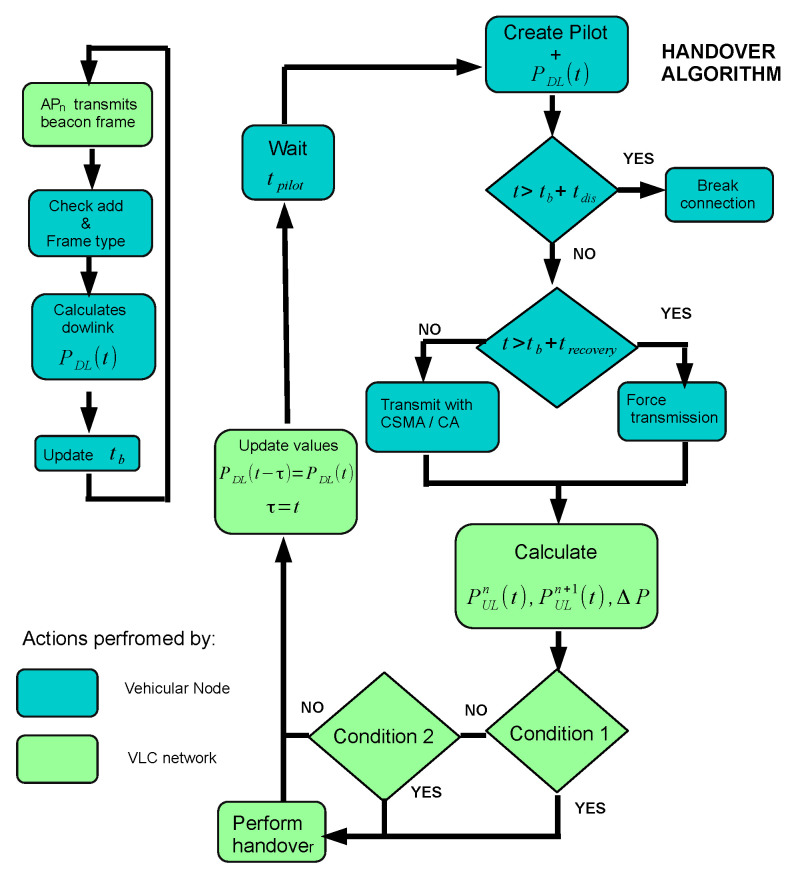
Diagram of handover decision algorithm.

This is followed by the handover decision stage, where the vehicular node updates the SNRs information and the system decides if such node is initializing a handover process. Figure 8 shows the flowchart of the handover decision algorithm that consists of two independent loops. The first loop updates the downlink information at the vehicular node when a beacon frame arrives from its associated coordinator. Then, it calculates $P_{DL}\left(t\right)$, and after that, it registers the reference time, $t_{b}$, of the transmitted beacon frame in the last synchronization process that helps to detect an outage. In the second loop, the vehicular node creates a pilot frame every $t_{pilot}=5$ ms, which includes the instantaneous SNR of the downlink $P_{DL}\left(t\right)$. Next, the vehicular node checks if the current time does not exceed the value of $t_{b}+t_{dis}$ with $t_{dis}$ as the threshold time of disconnection. Since the coverage area of two adjacent APs is not always overlapped, it is not possible to guarantee a soft handover. To consider this case, the system extends the disconnection time to allow the vehicle to cross this gap area by using the recovery time $t_{recovery}$. If the current time is greater than $t_{b}+t_{recovery}$ then the vehicular node forces the pilot transmission without using a CSMA/CA mechanism. The instantaneous SNR of the uplink channels are then calculated by those APs that receive the pilot frame, and only the value of $P_{UL}^{n+1}\left(t\right)$ is passed out to the HME entity.

To complete the handover decision stage, the HME entity checks the criteria of “condition 1”. It implies that $P_{UL}^{n+1}\left(t\right)>Th_{UL}$ and $P_{DL}\left(t\right)<Th_{DLmax}$, where $Th_{UL}$ is the instantaneous SNR threshold for the uplink channel, and $Th_{DLmax}$ the maximum instantaneous SNR allowed at the downlink channel. If “condition 1” is not satisfied, then the rate of change of $P_{DL}\left(t\right)$ is evaluated as a second criterion, *i.e.*

(6) $$\Delta P_{DL}=\frac{P_{DL}\left(t\right)-P_{DL}\left(t-\tau \right)}{\tau }$$

when the signal $P_{DL}\left(t\right)$ decreases below a certain threshold $Th_{DLmin}$ with a negative slope during the time interval τ, then the system will initiate the handover process. Note that the threshold estimates were obtained experimentally after a series of simulations. These values were $Th_{UL}=19$ dB, $Th_{DLmax}=75$ dB, and $Th_{DLmin}=50$ dB.

**Figure 9:**
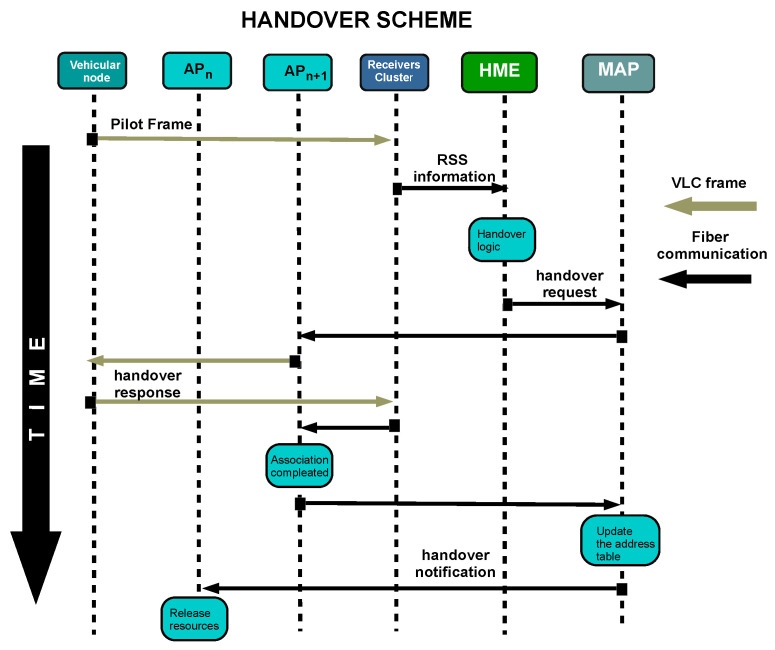
Handover execution protocol.

The last stage is the handover execution stage when the vehicle node switches over from its current AP to the next one. As mentioned before, the handover execution is based on HMIPv6. Figure 9 shows the execution scheme. After the gathering process stage, HME sends a handover request to MAP which checks if $AP_{n+1}$ has enough resources to receive an additional user. The request is delivered to $AP_{n+1}$ that tries to establish direct communication with the vehicular node. So, it emits the petition using the VLC channel. If the user can reply successfully, the handover completes its process, and $AP_{n+1}$ reports it to MAP. Finally, the $AP_{n}$ receives a notification that the user does not belong anymore to the network.

## 5. Evaluation Methodology

This section introduces the methodology and the simulations set up to evaluate the proposed handover scheme for high mobility users in a VLC network. During the vehicle trajectory, the system needs to detect when the vehicular node is leaving a cell to execute a handover. Our methodology uses a detailed representation of the MAC protocols and a realistic channel model to emulate the actual conditions of a V2I communication for a vehicle moving into a tunnel. It includes the use of the MMC method to simulate the channel impulse response, which takes into account the different transmitters’ radiation patterns, i.e., the vehicle’s headlamps and tunnel lamps. The evaluation setup is divided into three parts. First, we describe the methodology and the tools used, then the layout of the scenario and the parameters are introduced, and finally, we explain the metrics used for the performance evaluation.

We consider three hypothetical system configurations to validate the proposed handover solution:

- **Configuration 1:** Perform the handover process using only uplink information, i.e., satisfy the “Condition 1” criterion, in a conventional network topology.
- **Configuration 2:** Perform the handover process with the “Condition 1” criterion in the 2.5 layer network topology.
- **Configuration 3:** Perform the handover process using both uplink and downlink information, i.e., satisfy “Condition 1” and “Condition 2” criteria, in the 2.5 layer network topology, see Figure 8.

### 5.1. Network Simulation Tool

To emulate the real-time interactions of every vehicular node in the VLC network, a system-level simulation platform has been implemented. In this way, we can estimate the handover execution time since the system detects when a vehicle leaves a cell and until the next cell can communicate with this vehicle. The tool is designed based on the OMNet++ platform, which provides flexibility and modularity to the simulation model. With this approach, the vehicular nodes, and communication frames are represented by object-oriented components that enable to execution of the protocol decisions during the communication. Additionally, the network configuration is settled using a simple language named NED, which allows scaling the network size easily and automatizes multiple simulations.

**Figure 10:**
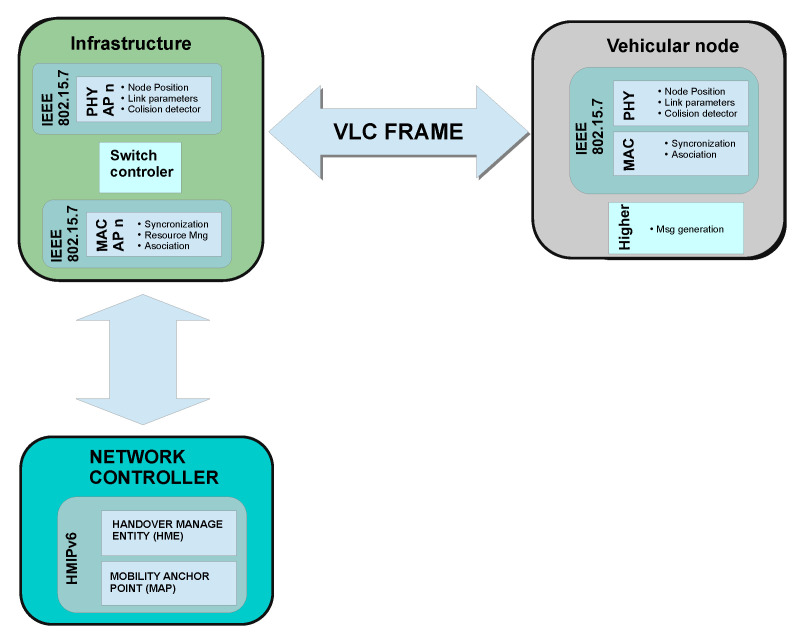
OMNet++ framework for handover simulation in VLC networks.

**Figure 11:**
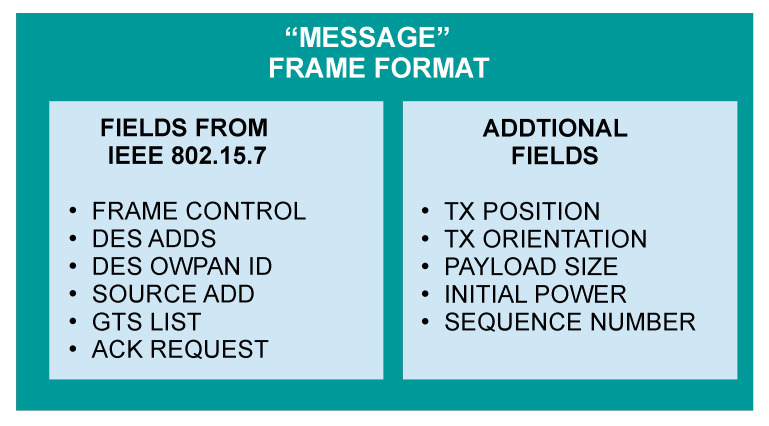
“Message” structure of the frame format in the simulation.

Despite OMNet++ helping to simulate the most common communication protocols such as INET, nowadays, there is not an open-source OMNet++ framework for VLC systems. For this reason, we needed to develop our component-based simulation libraries for the VLC framework in OMNet++. Figure 10 displays the simulation organization for the developed VLC network. It consists of three main modules: vehicular node, infrastructure module, and network controller module. Each module is composed of several subprograms to provide the signal propagation properties and the multiple logical processes. At the same time, the vehicular node and infrastructure module exchange information using a “message” object with the most relevant fields of the frame format defined in the IEEE 802.15.7 standard. Thus, we have also developed a special object-oriented program called “Message” that symbolizes the frame transmission and permits interaction between these components. Figure 11 shows the “message” frame format used in the simulations. Additionally, this message has extra information for providing more realistic simulations.

We have organized the vehicular node and infrastructure module into two components: PHY and MAC layers. While the PHY layer evaluates packet transmissions, the MAC layer performs the logical decisions according to the frame control information of each message. Therefore, the proposed network simulator requires a set of physical layer modules connected to a controller to simulate the network receivers’ distribution. This controller will filter the redundant frames and send the frame representation message to the corresponding MAC layer submodule. Since the channel access and other controls are managed individually, they require an individual logical decision submodule. Consequently, the infrastructure module consists of the same set of MAC layer submodules joined to the controller. They will receive the frame and resolve the corresponding protocol action.

**Figure 12:**
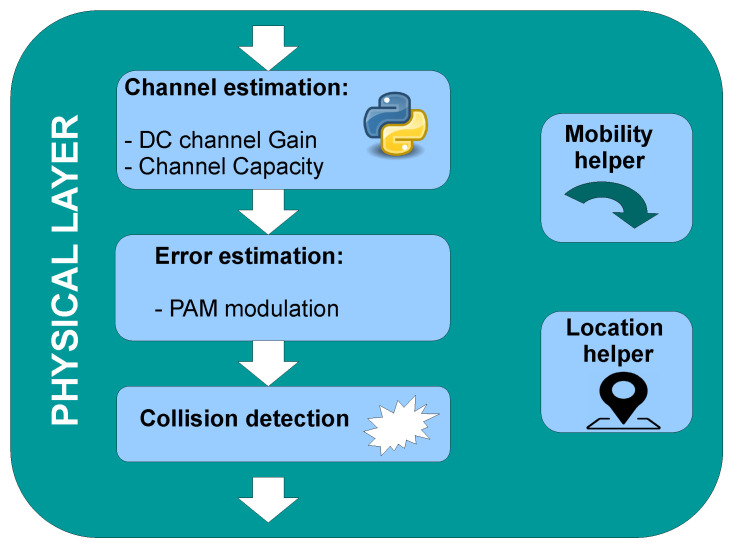
Physical layer submodule.

The PHY layer submodule evaluates the VLC link status and channel properties to determine when it is physically possible to receive a frame. The same PHY layer submodule is implemented both in the vehicular node and the AP node. Figure 12 shows the internal organization of the PHY layer block. It only takes care of the signal transmission, so it processes all incoming frames without segregating or ignoring them by their header information (OWPAN ID or destination address). First, the transmitter puts a stamp in the “message” frame with its spatial information. When any node receives this “message” it calculates the channel impact on the transmission using MMC with 50,000 random rays and 3 rebounds each. MMC is done considering the specific radiation pattern of both light sources (tunnel streetlights and vehicle headlights), they are shown in Figure 12. Both patterns differ from the typical Lambertian profile and were modeled using the technical information of tunnel streetlamps and experimental measurements from a car headlight. Additionally, the reflection coefficient from concrete and asphalt were considered with a value of 0.17 and 0.07 respectively [59].

The information from the MMC is then used to assess the DC channel gain and the RMS delay spread which are associated with the received power and bandwidth restrictions, respectively. This channel evaluation requires the current node’s position which is obtained through this submodule. After that, the bit error rate and frame error rate are computed using the frame length, the received power, and the modulation scheme information. With these probability models, the PHY layer submodule decides when a frame transmission was successful. Finally, the submodule retains the packet to assure that the communication is not interrupted by another transmission. If two transmissions happen during the signal propagation time, a collision occurs.

On the other hand, the MAC layer submodule executes the logical decisions. When a frame arrives at this submodule, it verifies the OWPAN ID or destination address to be sure that it needs to process this frame. Also, it checks that the transmission was done in the appropriate communication window inside the superframe structure. Then, it executes the corresponding action for the frame control information in the header. Additionally, this submodule performs the channel access scheme and other protocols of the MAC layer from the standard IEEE 802.15.7 as described in Section 3.1. A deeper description of this simulator structure can be found in [60].

Meanwhile, the network controller is divided internally into two submodules: MAP and HME. MAP registers any user in the network, and HME checks if the user needs to perform a handover. Furthermore, the network controller sub-module also generates some objects that represent the data frames on the network. These frames are sent to the user using the transmission protocols simulating in the V2I communication. These packet representations help to evaluate the network performance and other metrics as those described in Section 5.3.

### 5.2. Experimental Setup

**Figure 13:**
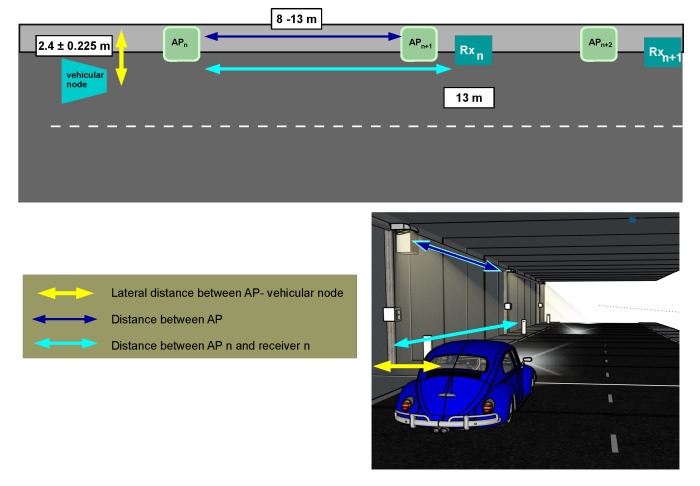
Scenario 1: Graphical representation of the VLC network with one vehicular node.

Our proposed VLC network topology and handover protocol for high mobility is evaluated by simulations in two different scenarios. The first scenario measures and compares the performance of the three configurations considering a single user. While the second scenario selects the configuration with the best performance. Then, it evaluates the protocol’s execution when the network increases its user number. Figure 13 shows the graphical representation of the first scenario. It considers a single-vehicle moving at 80 km/hr through a two-lane tunnel with a lighting system that contains a set of luminaries (APs). The enclosure has the maximum regulatory dimensions with a width of 13.2 m and 4.9 height [61]. The luminaries usually are mounted on the wall at 4.2 m above ground level with an elevation angle of 20°. We remark that the lamp positions follow the existing norm of tunnel-lighting in most countries [62], which recommends 8 to 13 m between two luminaries to avoid generating flickering effects to the drivers. Therefore, our configuration proves feasible in real scenarios by reusing the hardware of the existing illumination systems. On each AP, the VLC receiver is 13 m forward of the luminary mounted on the wall and 50 cm above the road, opposite to the traffic direction with a 90° elevation, to ensure a good signal reception over an adequate period. On the other hand, the vehicular node has a built-in VLC system, which uses its headlamps as the transmitter and adds a receiver near to the left headlamp (on the hood), pointing to the tunnel ceiling with an elevation of 180°. The vehicular node is assumed to be transiting on the lane closest to the tunnel wall with the transmitter (headlamps) at 1 m above the ground level and its receiver at 2.4 m from the tunnel wall. This is the major distance between the lane edge and the closest wall in a two-lane tunnel. Consequently, the critical scenario for the uplink. Since the natural driving of a person makes the car moves from side to side in its lane, it loses its alignment with the road lane and consequently with the tunnel wall. Therefore, the handover process may be affected by these displacements, but also the narrow radiation pattern of the headlamps. For these reasons, we include an error margin of 0.225 m to the vehicle’s position against the tunnel wall to simulate these types of behaviors. For the simulations, the initial position of the vehicular node starts from 10 to 13 m behind the first tunnel luminary. This configuration evaluates the vehicle’s communication with a receiver placed in the closest wall. Additionally, this simulation setup assumes that the vehicles keep a safe separation distance, providing Line-of-Sight communications at any time.

The simulation process is performed as follows. First, the vehicular node moves straight forward with a steady speed while the APs are transmitting beacon frames. When the vehicular node receives one of these frames, it starts the discovery process to find the best possible connection. Then, it waits for a random period and then sends an association request. After the association process is resolved, the AP transmits data frames with a constant throughput. When the vehicle is leaving the AP coverage area, the network needs to detect its departure using the handover decision algorithm described before. If the estimation is correct, the handover protocol is executed to start a new session in the next AP and continue the communication. In other cases, the handover process fails, the AP will continue transmitting the data frames until the disconnection time expired. At the same time, the vehicular node detects a failure if it stops receiving the beacons. Then, the vehicular node needs to keep sensing the channel looking for an available AP to start the association process again.

**Figure 14:**
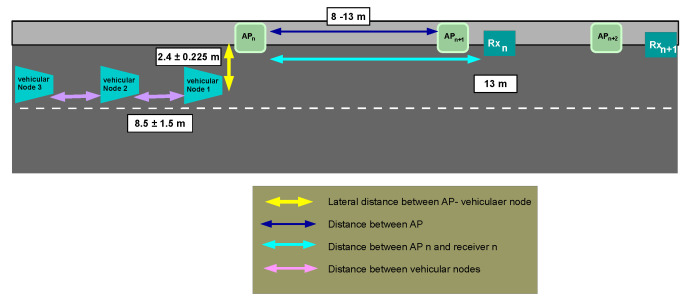
Scenario 2: Graphical representation of the VLC network with multiple vehicular nodes.

The second scenario considers vehicles in a convoy that commute in a tunnel, see Figure 14. For this scenario, we extend the experimental evaluation of the handover process which can be depleted by vehicles sharing the same channel. In this setup, there are *m* vehicles moving at the same speed as a caravan. The infrastructure configuration is the same as the single-vehicle evaluation. And the leader vehicles are placed in the same position too. The distance between subsequent vehicles is defined as a random variable uniformly distributed over the range of 7 to 10 m. This scenario does not accomplish the suggested safety separation distance between vehicles, but it is done to evaluate the possible case when a vehicle ingresses an occupied cell. Under this situation, the vehicular nodes need to share resources. Therefore, there is a possibility that one of the handover messages may collide with another vehicle’s communication. Other relevant changes in the experimental configuration include the vehicular nodes’ position, the network’s conditions, the number of nodes connected to a particular AP, etc. The simulation process of this second scenario follows the same process as described in the single vehicular node case.

Table 2 summarizes the more relevant simulation parameters for the experimental scenarios. Each experiment was evaluated 100 times under the same parameters. The parameters considered has been 8 different distances between AP, 3 system configuration (1 start topology, and 2 proposed topology), and 2 scenarios (single-user and multiples users). There is a total of 2400 simulations for the first scenario, and 3200 simulations for the second scenario. Each simulation recreates 18 s of communication where the vehicle needs to perform between 27 to 50 handovers to have seamless communication. The decision algorithm is executed when a vehicle sends a pilot every 5 ms, it implies that the detection algorithm is executed around 3600 times in a single simulation. Additionally, the nodes update their channel every 1 ms to have smooth changes and have an accurate model.

The communication system follows the IEEE 802.15.7 specs using a 60 MHz optical clock with an OOK modulation in the PHY II mode. Moreover, the separation range between the APs is 8 to 15 m, the VLC transmitter powers of the tunnel luminary and the vehicular nodes’ headlamp are 50 W and 15 W respectively, and an active surface area of the VLC receiver of 1 mm^2^ and Field-of-View of 60°. For this initial evaluation, there is not considered the integration of a lent in the receiver.

**Figure 15:**
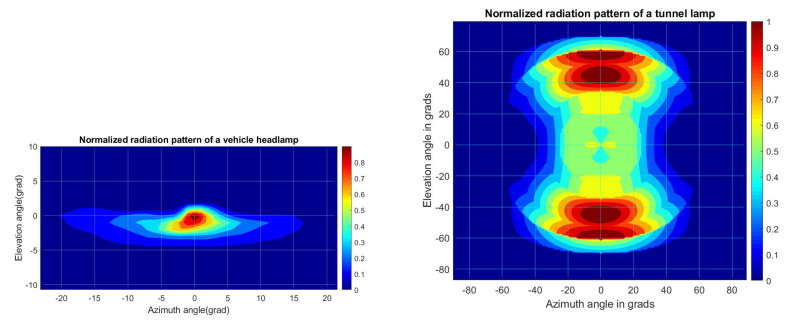
On the left side, the headlamp radiation pattern, and on the right the tunnels lamp radiation pattern.

Additionally, some MAC parameters were set up. Firstly, BI was set at 2 ms. This time is long enough to allow two or more users to share the same medium but not so prolonged in case they lose synchronization. Such time could be fixed by adjusting B0, SO, and aBaseSuperframeDuration values, as can be seen on the parameters table. Also, the channel access mechanism was set to execute up to 5 additional transmissions tries before it discards the information. In each new attempt, the distribution time for the random access increases exponentially using a aBackofUnit base of 200 in its calculation. The simulation considers a frame data structure that comprises a payload size of 1000 bits with a header of 207 bits. The header size is an approximation considering the bits in each one of its fields. The payload for beacon and signaling frames was fixed to 500 bits. The frame is encoded with Run-length limiting (RLL) which increases the frame size with a ratio of 10 bits per 8 input bits (8b10b), these aspects determine the transmission period. The value of Radiation Pattern is shown in Figure 15.

Finally, the following parameters were established for the handover process: the disconnection time $t_{dis}$, recovery time $t_{recovery}$, association latency $t_{a}$, and the handover latency $t_{h}$. They determine the latency of the handover and the outage duration when this process fails. A vehicular node will lose synchronization when it has not received a beacon frame after $t_{recovery}=40$ ms, then it will be disconnected after $t_{dis}=300$ ms. This tolerance period allows the vehicle to do the handover process when the network has a gap area between two APs.

### 5.3. Performance Analysis

The following metrics are used to measure the performance of the proposed vehicular VLC network. The first metric is the service availability which is calculated by using the following equation:

(7) Ratio=(servicetime−interruptedtime)servicetime

Availability is measured as the ratio of time that the vehicular node’s communication service is available. The node requires to receive and transmit data successfully to be considered with communication service. The monitoring period is set to 5 ms, and if the user receives at least one frame, the node is successfully connected in this interval. Otherwise, the system assumes that the node has lost connection during this period. The service time starts when the user has received its first data frame. Repeated frames are ignored because it means that the node is not able to reply to the communication.

The second metric is the End-to-End Frame Error Rate (EFER) measurement. It represents the reliability of the system after the re-transmission and failure detection mechanism of the MAC layer. A frame can be lost if the re-transmission limit time is exceeded and the AP is not able to reach the node. Therefore, the data packet transmission performance can be characterized as the ratio of the incoming data frames from upper layers in a particular AP, referred to as “total frames”, and the transmitted frames which have been received correctly in the MAC layer of the vehicular node, referred as “delivered frames”, i.e.,

(8) EFER=1−deliveredframestotalframes

The third metric is the Frame Error Rate on the PHY layer (PFER). This measure considers all the AP transmission attempts, referred to as “the number of transmissions”, including the transmitted frames which were not correctly delivered. This will reflect the total spent resources during the communication. In a similar fashion to EFER, PFER uses the complement of the ratio between delivered frames and the total number of transmission frames,

(9) PFER=1−deliveredframesnumberoftransmissions.

PFER and EFER require a bidirectional communication to increase the number of “delivered frames”, including the successful transmission with an acknowledgment frame.

Finally, the average data rate of the vehicular node is also measured to evaluate the performance of the network service quality. The instantaneous data rate metric, $R_{ins}$, is defined as

(10) $$R_{ins}=\frac{\sum_{i=1}^{rf}P_{load}\left(i\right)}{t_{m}}$$

where $R_{ins}$ is the data rate measured during the time window $t_{m}=5$ ms with $P_{load}\left(i\right)$ as the payload of the *i*-th received frame. Then, the average data rate, $R_{avg}$, during the total simulation time, $t_{sim}$, is given as

(11) $$R_{avg}=\sum_{j=1}^{t_{s}/t_{m}}\frac{t_{m}R_{ins}^{j}}{t_{s}}$$

## 6. Results and Discussion

This section presents and analyzes the results of the network performance for the different setups. Firstly, we study the single-user case scenario. Then, it explores the network with multiple users. Finally, we discuss if the expectations of the system performance have been accomplished.

### 6.1. Single Vehicle

**Figure 16:**
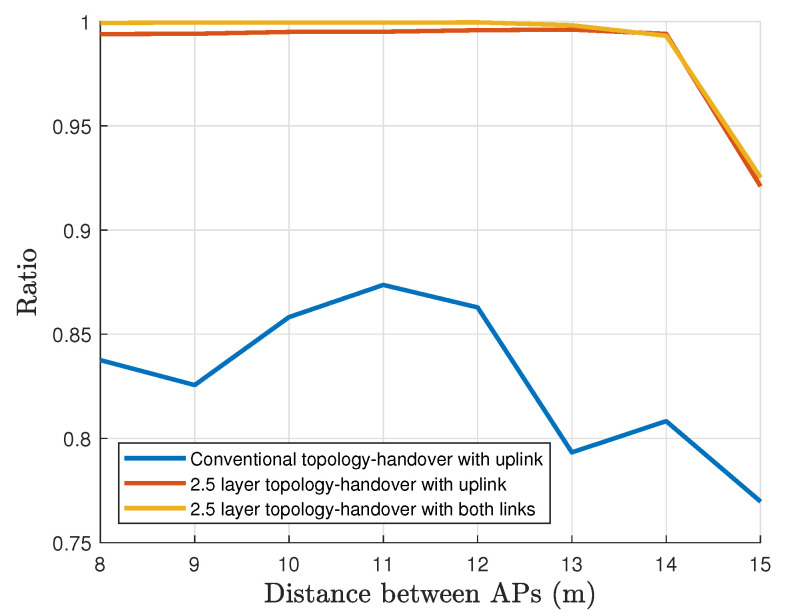
Service availability comparison with single user.

This evaluation contemplates a single vehicular node under a controlled environment. Figure 16 shows and compares the service availability, defined in Equation (7), as a function of the APs separation under the three different scenario configurations described in Section 5. We observe that configurations 2 and 3 keep a high availability when the separation between the APs is less than 14 m. For a higher separation, the service availability begins to decay. With configuration 1, this service declines below 85% at a distance of 8 m and gets worse for larger distances. It is clear that the proposed 2.5 layer topology yields a significant improvement in the link reliability as compared with the conventional topology. The main reason for this behavior is that in a conventional topology the link can be broken before the handover starts, which causes to stop gathering information to execute the handover.

**Figure 17:**
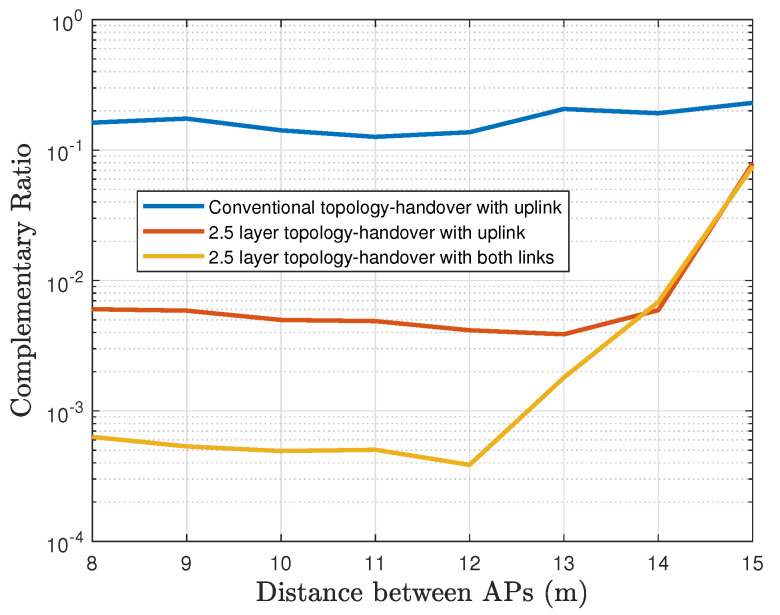
The outage ratio comparison (logarithmic scale).

Figure 17 provides the service time ratio using a logarithmic scale. The best overall performance is achieved with Configuration 3. We see that the interruptions in Configuration 2, using only the uplink information, are ten times longer than the interruptions in Configuration 3, where the service is interrupted averagely 1% of the time while it only uses the uplink information. Otherwise, this interruption time decreases until 0.1% in Configuration 3. It is not always possible to make the handover when the distance that separates the APs is more than 12 m, therefore, the performance decreases. We highlight that the 2.5 layer topology can support communication even when the link has been broken, as a result, it provides a supplementary communication time to detect the movement, and as consequence, there is seamless communication without severe interruptions. This improvement guarantees that the vehicle will stay connected almost all the time.

**Figure 18:**
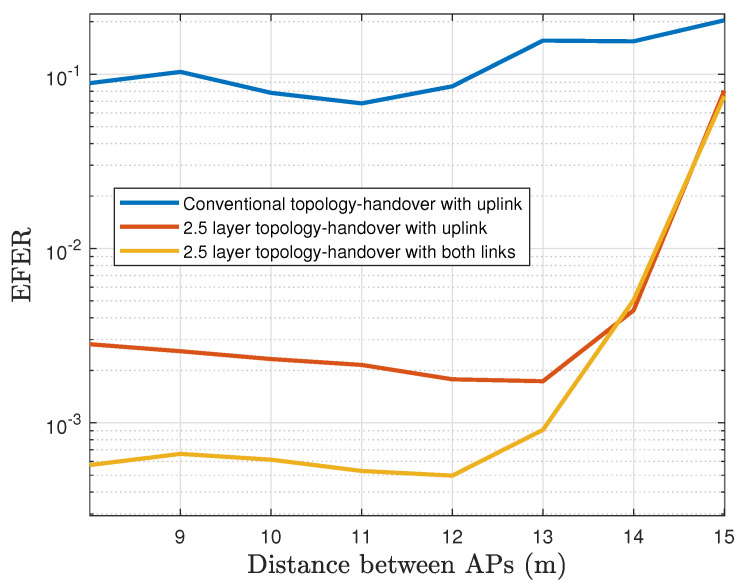
End-to-end frame error rate considering the backoff mechanism (logarithmic scale).

The measured EFER of the VLC network with a single vehicular node is now shown in Figure 18. Here, we observe that Configuration 1 delivers EFER rates of about 10^−1^ which are considered too high for vehicle safety messages. Configuration 2 achieves a performance of EFER rates lower than $3\times 10^{-3}$ when the separation between APs is under 13 m, then it increases up to $7\times 10^{-2}$ for separation of 15 m. Finally, Configuration 3 shows the most favorable performance with an EFER of $5\times 10^{-4}$, almost one order of magnitude higher than Configuration 2. From a separation of 12 m between APs, the performance begins to decline, and eventually, Configurations 3 and Configuration 2 obtain the same results.

**Figure 19:**
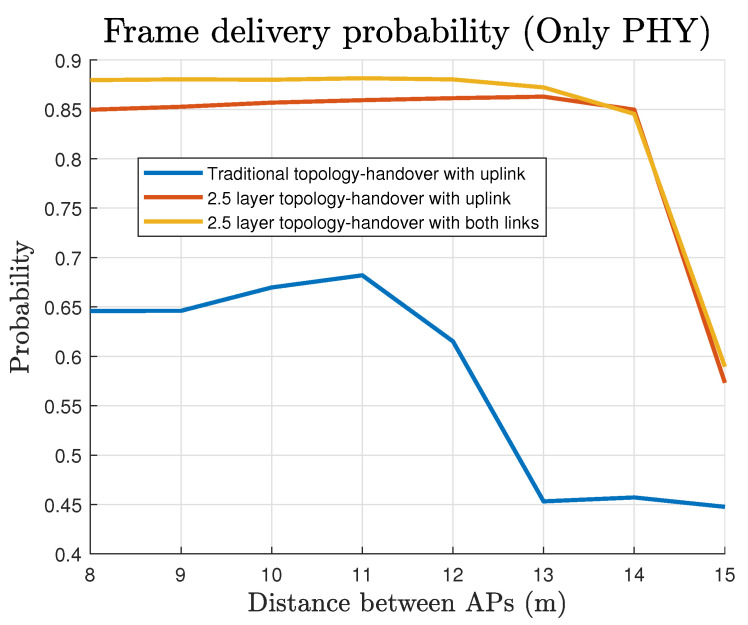
Physical layer Frame Error Rate (logarithmic scale).

Similarly, in Figure 19 the compared PFER is presented concerning the position of the AP. From these results, the maximum errors for Configuration 2 and 3 do not exceed 15% in the case where the separation between the APs is below 14 m. Configuration 3 shows the minimum PFER of about 12% up to a distance of 13 m. On the contrary, the successful transmissions with the conventional topology reach a PFER of about 0.65 for distances below 12 m, and for longer distances between APs, the received frames are reduced to half of them.

**Figure 20:**
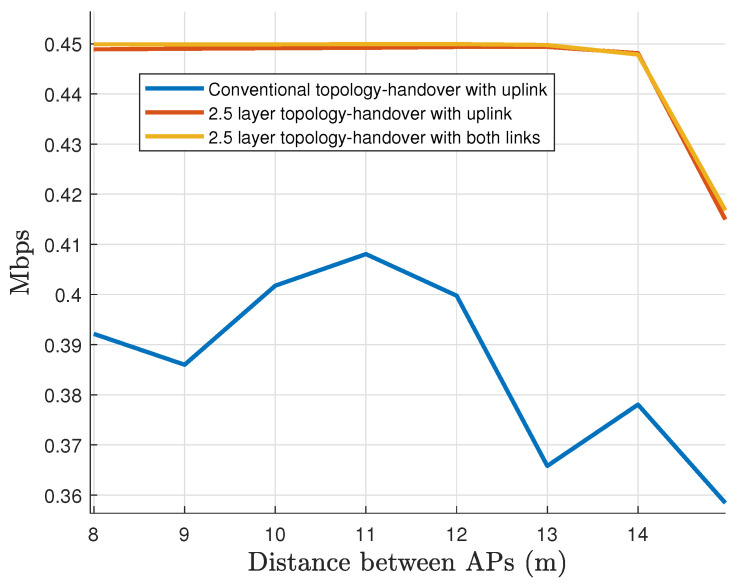
Average User’s data rate during the entire simulation.

Finally, we plot the behavior of the data rate service in the Figure 20. The network is set to transmit a steady data rate of 450 kbps per vehicular node that proves to be enough to hold message-based safety applications as suggested in [63]. These results reflect that there is not a substantial throughput difference between Configurations 2 and 3. The system can handle the maximum requirements for both configurations thanks to the redundancy of the uplink channel. On the other hand, Configuration 1 suffers from throughput degradation problems because of the lack of redundancy.

### 6.2. Multiple Vehicles

One of the goals of a handover algorithm is to prevent a handover failure due to insufficient resources in the target cell. To measure this influence, we evaluate the performance of the network considering a scenario with multiple vehicles as described in Section 5.2. For these evaluations, we compare only for configuration 3 the service availability, PFER, and EFER, as the number of vehicular nodes increases.

Moreover, due to the limited cell coverage, the individual user performance is not impacted deeply by scaling the network with more users. It means that we do not have to scale up the simulation with a large number of vehicular nodes to determine the performance limit. Furthermore, the coverage area of each AP helps to delimit to a maximum of 3 vehicles per cell, minimizing the need of serving a large number of vehicles.

**Figure 21:**
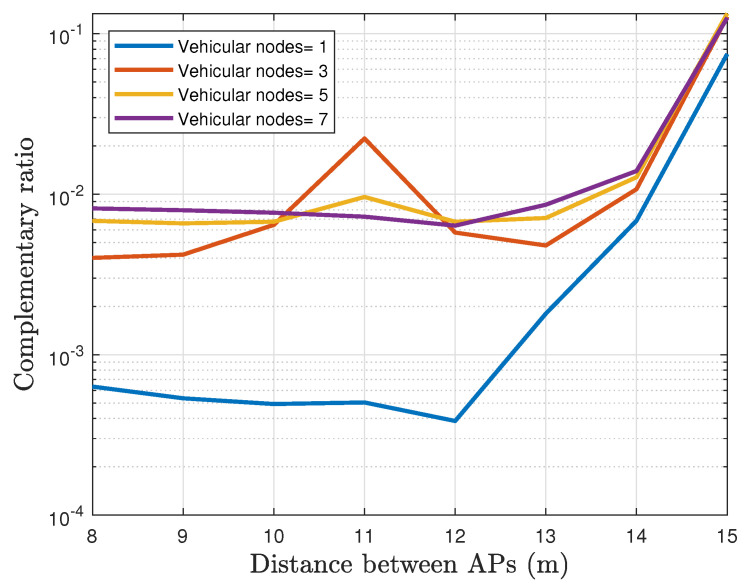
The complement of the service availability for different number of vehicular nodes operating in the network (Outage ratio).

From Figure 21, it is evident how the service availability decreases with the number of vehicular nodes. For this scenario, the service time is affected because multiple vehicles join the same AP. Therefore, the AP does not transmit any information to a new vehicle until the previous frames in the buffer of the AP have been delivered. Thus, the service is more likely to be interrupted during the handovers. As expected, the results depicted in Figure 21 corroborate how the service time is reduced on average. Moreover, the operation of multiple vehicles in a close area makes the blackout time increase up to 20 ms when the AP separation distance is less than 13 m. Furthermore, when the separation of the APs is shorter, the handover occurs much more frequently.

**Figure 22:**
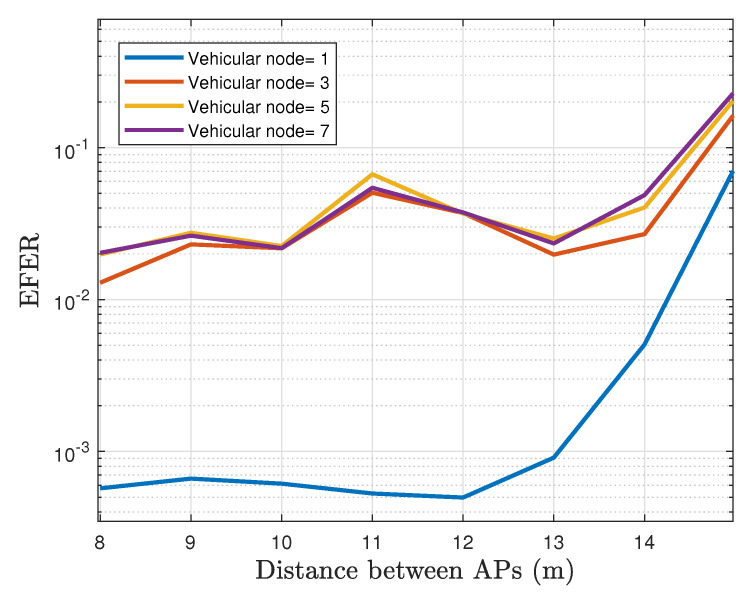
The EFER for different number of vehicular nodes operating in the network.

**Figure 23:**
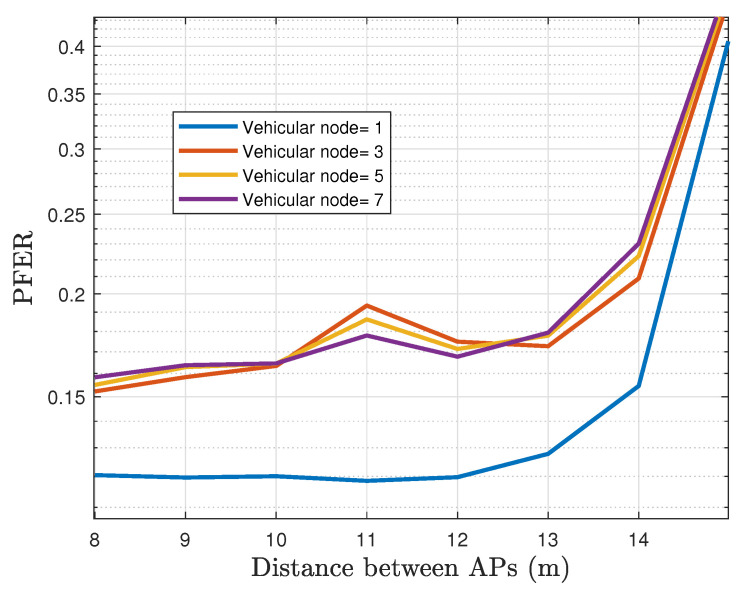
The PFER for different number of vehicular nodes operating in the network.

Figure 22 plots the comparison of the EFER for a different number of vehicular nodes. The results exhibit a considerable drawback when more than one user is associated with an AP. We observe that the EFER converges to $3\times 10^{-2}$ at distances under 13 m, which is a substantial rise in comparison with $5\times 10^{-4}$ from the single-vehicle scenario. On the other hand, Figure 23 shows that the PFER performance has a similar tendency to EFER. As expected, a single-vehicle crossing the tunnel achieves superior performance with an error rate of 12% when the distances between lamps are less than 12 m, which is regulatory. However, PFER does not exceed a maximum value of 19% regardless of the number of vehicles in the same scenario. Indeed, the PFER performance value for three or more vehicles does not change notably.

**Figure 24:**
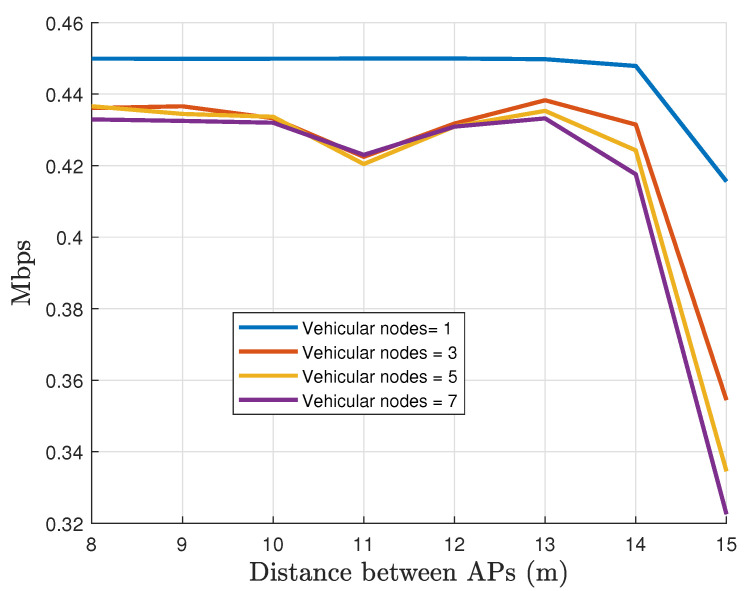
The data rate per vehicular node for different number of vehicular nodes operating in the network.

Finally, Figure 24 depicts the measured data rate with respect to the separation of the AP and the number of active vehicular nodes. Here, the data rate is reduced to 420 Kbps with *m* = 3, 5, and 7 vehicular nodes in the network. More interesting, it is worth noting how quickly the data rate of convergence is obtained when the separation between APs is under 14 m. It means that the solution is scalable without significant penalties.

### 6.3. Discussion

Our experimental simulations evaluate the performance of a vehicular VLC network in a tunnel scenario for single and multiple vehicles. The results provide further knowledge about the behavior of the VLC network in realistic use-case scenarios for vehicular users and a promising proposed handover mechanism that copes with high mobility. However, the network’s performance can be affected by the number of users due to the shared medium. But, this is a common issue that both technologies have to deal with (VLC and DSRC). It is demonstrated in [64] that DSRC suffers similar drawbacks when the vehicle density grows. The packet delivery ratio diminishes in DSCR under these conditions. Moreover, a fair comparison of the performance degradation for these two technologies is difficult due to their remarkable differences. On the one hand, VLC cell does not require sharing resources with multiple vehicular nodes, while DSRC cell provides service to a wide area. Thus, it has a channel used by a large number of nodes. On the other hand, a VLC network will require more frequent handovers to provide service. This action will also limit the service quality.

Despite its disadvantages, the proposed VLC handover strategy appears as a promising scheme to support communication for some vehicular scenarios. As mentioned before, these are initial studies with several issues that remain unsolved. In this work, we designed a realistic vehicular tunnel scenario to evaluate the operation of the proposed VLC network. However, it is not a straightforward process to adapt our design to other environments. For example, the detection algorithm is simple and takes advantage of the scenario geometry and the limited vehicle’s degree of freedom. So, this algorithm can not be used in other urban scenarios, such as streets.

On the other hand, the 2.5 layer topology is a solution that has the potential to solve the communication in other indoor scenarios, such as offices. Finally, the developed simulation platform can evaluate some other mobility scenarios for VLC users. For instance, a vehicle moving through a random trajectory or a VLC gadget operating inside an office. Although, it is necessary to design detection strategies for each.

## 7. Conclusions

In this paper, we have explored an encouraging VLC network solution to support communication in vehicular tunnels. The performance of the proposed solution has been studied through a system-level simulator that validates the functionality of an operating VLC network instead of peer-to-peer communication. As a result, this study provides a better understanding of the challenges VLC networks face to support the implementation of vehicular communication. These early findings, though promising, will need further development to assist vehicular communications with high mobility.

The method is exemplified in a vehicular VLC network that operates over a tunnel. This specific scenario is employed to examine the proposed solution consisting of a network topology and a handover mechanism that achieve lower handover latencies. When the vehicle goes through a tunnel, the communication service is barely interrupted under a controlled environment. In the worst-case scenario, the interruptions do not span more than 20 milliseconds. Additionally, the quality of service is not affected during the vehicle trajectory where the communication link remains under a steady data rate and FER below 10^−3^. These results demonstrate that the system can provide trusted services to deliver emergency notifications, crucial for Automated Guided Vehicles. Furthermore, the proposed solution also applies to urban scenarios, where the number of vehicular nodes per area is high. This factor does not impact the system performance considerably.

As mentioned previously, the VLC network design for vehicular communication is still in the early stage of its development. Future works include studies of more complex scenarios, for example, considering interferences from vehicles in different lanes and changing their speed dynamically. Also, it is necessary to explore other cases when the vehicles can cause an obstruction. For example, in a road tunnel with more lanes, the uplink of a vehicle transiting in the central lane can be blocked by other vehicles. However, we would require to extend the simulator capabilities to evaluate these scenarios. Moreover, the propagating optical channel can suffer from some degradation effects that need incorporating in the simulations, such as car fume, dust, and turbulence effects. The handover estimation can be more reliable by adding supplementary tracking mechanisms to deal with erratic movement patterns. Eventually, it will be necessary to enhance the system design and perform practical trials to validate the solutions.

## Figures and Tables

**Table 1:** Parameters for the SNR calculation.

Parameter	Value	Parameter	Value
$R_{\lambda }$ Resposivity	0.2 A/W	*q* electron charge	$1.60217e^{-19}$ C
$i_{d}$ receiver dark current	0.562	$I_{B}$ background current	$1.13e^{-6}$ A
*B* Noise Bandwidth	300,000 b/s	$i_{d}$ dark current	10 nA
G Open Loop Voltage Gain	10	η Fixed capacitance	112 pF/cm^2^
$T_{k}$ Temperature absolute	298	Γ FET channel noise factor	1.5
$I_{3}$ Noise Bandwidth Factor	0.0868	$g_{m}$ FET transconductance	30 mS
$I_{2}$ Noise Bandwidth Factor	0.562	*k* Boltzmann’s Constant	$1.3806e^{-23}$ J/K

**Table 2:** Simulation parameters.

Paramaters	Value	Parameters	Value
Phy Layer			
Optical Clock	60 MHz	Headlamp power	15 watts
AP Separation	8–15 m	Tunnel Lumminary power	50 watts
Vehicel speed	80 km/h	Receiver area	1 mm^2^
Modulation	OOK	Run-length limited	8b10b
PHY mode	II	Forward Error Correction	none
Rx FOV	60°	Lens	No
Radiation Pattern	See Figure 15	MMC number of ray	50,000
Reflection coefficient concrete	0.17	Bound per ray	3
Reflection coefficient asphalt	0.07	Channel update	1 ms
MAC Layer			
Frame Header	207 bits	BO, SO	5,7
Signaling message size	500 bits	User Throughtput	450 Kbps
aBackoffUnit	200	Frame payload	1000 bits
aBaseSuperframeDuration	60	Number of backoff max	5
Handover			
THDL	19 dB	Recovery time	40 ms
THUL max	75 dB	Disconnection time	300 ms
THUL min	50 dB	Association latency	200 ms
Time-to-Trigger	10 ms	Handover latency	2 ms

## Data Availability

Not applicable.
